# Nutrient/serum starvation derived TRIP-Br3 down-regulation accelerates apoptosis by destabilizing XIAP

**DOI:** 10.18632/oncotarget.3112

**Published:** 2015-02-21

**Authors:** Chengping Li, Samil Jung, Soonduck Lee, Dongjun Jeong, Young Yang, Keun-Il Kim, Jong-Seok Lim, Chung-Il Cheon, Changjin Kim, Young-Sook Kang, Myeong-Sok Lee

**Affiliations:** ^1^ Department of Life Systems, Sookmyung Women's University, Seoul, 140–742, South Korea; ^2^ Department of Pathology, College of Medicine, Soonchunhyang University, Chonan, 330–090, South Korea; ^3^ College of Pharmacy, Sookmyung Women's University, Seoul, 140–742, South Korea

**Keywords:** Nutrient/serum starvation, apoptosis, TRIP-Br3, TRIP-Br1, XIAP

## Abstract

TRIP-Br3 and TRIP-Br1 have shown to have important biological functions. However, the function of TRIP-Br3 in tumorigenesis is not well characterized compared to oncogenic TRIP-Br1. Here, we investigated the function of TRIP-Br3 in tumorigenesis by comparing with that of TRIP-Br1. Under nutrient/serum starvation, TRIP-Br3 expression was down-regulated slightly in cancer cells and significantly in normal cells. Unexpectedly, TRIP-Br1 expression was greatly up-regulated in cancer cells but not in normal cells. Moreover, TRIP-Br3 activated autophagy while TRIP-Br1 inactivated it under serum starvation. In spite of different expression and roles of TRIP-Br3 and TRIP-Br1, both of them alleviate cell death by directly binding to and stabilizing XIAP, a potent apoptosis inhibitor, through blocking its ubiquitination. Taken together, we propose that TRIP-Br3 primarily activates the autophagy and suppresses apoptosis in nutrient sufficient condition. However, the prolonged extreme stressful condition of nutrient starvation causes a dramatic decrease of TRIP-Br3, which in turn induces apoptosis by destabilizing XIAP. Up-regulated TRIP-Br1 in cancer cells compensates this effect and delays apoptosis. This can be explained by the competitive alternative binding of TRIP-Br3 and TRIP-Br1 to the BIR2 domain of XIAP. In an extended study, our immunohistochemical analysis revealed a markedly lower level of TRIP-Br3 protein in human carcinoma tissues compared to normal epithelial tissues, implying the role of TRIP-Br3 as a tumor suppressor rather than onco-protein.

## INTRODUCTION

Many human malignancies, including cancers, are caused by imbalance between cell death and survival. Considering the facts that deregulation in cell death causes cancer and in turn cancer cells aggravate tumorigenesis by increasing a resistance to cell death, the elucidation of the molecular pathways used in the regulation of cell death is very important. Cancer cell death can be caused by many different types of stresses. One of them is extreme nutrient deficiency. Unlike normal cells, cancer cells are often placed under considerable nutrient stress because of their uncontrolled growth and proliferation. Thus, cancer cells are much more sensitive to cell death than normal cells in low-nutrient environment. However, many cancer cells are found to have acquired a tolerance for nutrient depletion derived cell death during tumorigenesis. Tolerance to nutrient depletion is very important for cancer cells especially at the early stage of tumorigenesis and during invasion or metastasis. It is because cancer cells need more time for the development of the regional angiogenesis to supply nutrients for the growth of cancer cells. Considering these facts, the better understanding of the molecular mechanism of nutrient-independent cancer cell growth seems to be very important in cancer research. Eukaryotic cells have evolved various cellular processes including autophagy and apoptosis in response to nutrient starvation. Nutrient starvation causes autophagy to provide cells with additional internal nutrient supplies [1–6]. However, further extreme nutrient deficiency due to prolonged starvation eventually induces apoptosis [5–8]. Our study suggests that TRIP-Br3 and TRIP-Br1 seems to have important roles in this process.

TRIP-Br family has been involved in multiple biological functions in cells, such as transcriptional regulation, cell cycle, apoptosis, tumorigenesis, metastasis and metabolism [9–18]. The TRIP-Br family includes TRIP-Br1 (also known as SERTAD1/SEI-1/p34^SEI-1^), TRIP-Br2 (SERTAD2/SEI-2), TRIP-Br3 (SEI-3/CDCA4/Hepp), and SERTAD3 (RBT1/SEI-4). TRIP-Br members share five conserved regions with a high degree of homology: CyclinA binding domain, SERTA motif, PHD/bromo domain interacting domain, transcription activating domain, and NES (Nuclear export sequence) [19–22]. However, each member has been detected in different sub-localization with different levels [19] implying their similar but different cellular functions. In fact, it was proposed that TRIP-Br3 negatively regulates tumorigenesis by inhibiting the expression of JUN oncogene, while TRIP-Br1, TRIP-Br2, and SERTAD3 positively affect it in multiple human cancers [9, 12, 21, 23–28]. Furthermore, TRIP-Br3 inactivates the transcription of E2F1 responsive genes, while other members activate it [9, 21, 25–27]. Thus, TRIP-Br3 seems to exert different functions from other TRIP-Br members in cells even though they belong to the same family. In the effort of elucidating the resistance of cancer cells to nutrient starvation induced cell death, we found the TRIP-Br3 down-regulation and TRIP-Br1 up-regulation in response to nutrient starvation. In this report, we show how TRIP-Br3 regulates cell death coordinately with TRIP-Br1 in cancer and normal cells under the nutrient deficient conditions.

## RESULTS

### Down-regulated TRIP-Br3 gene expression in a variety of cancer and normal cells under nutrient starvation

**Figure 1:**
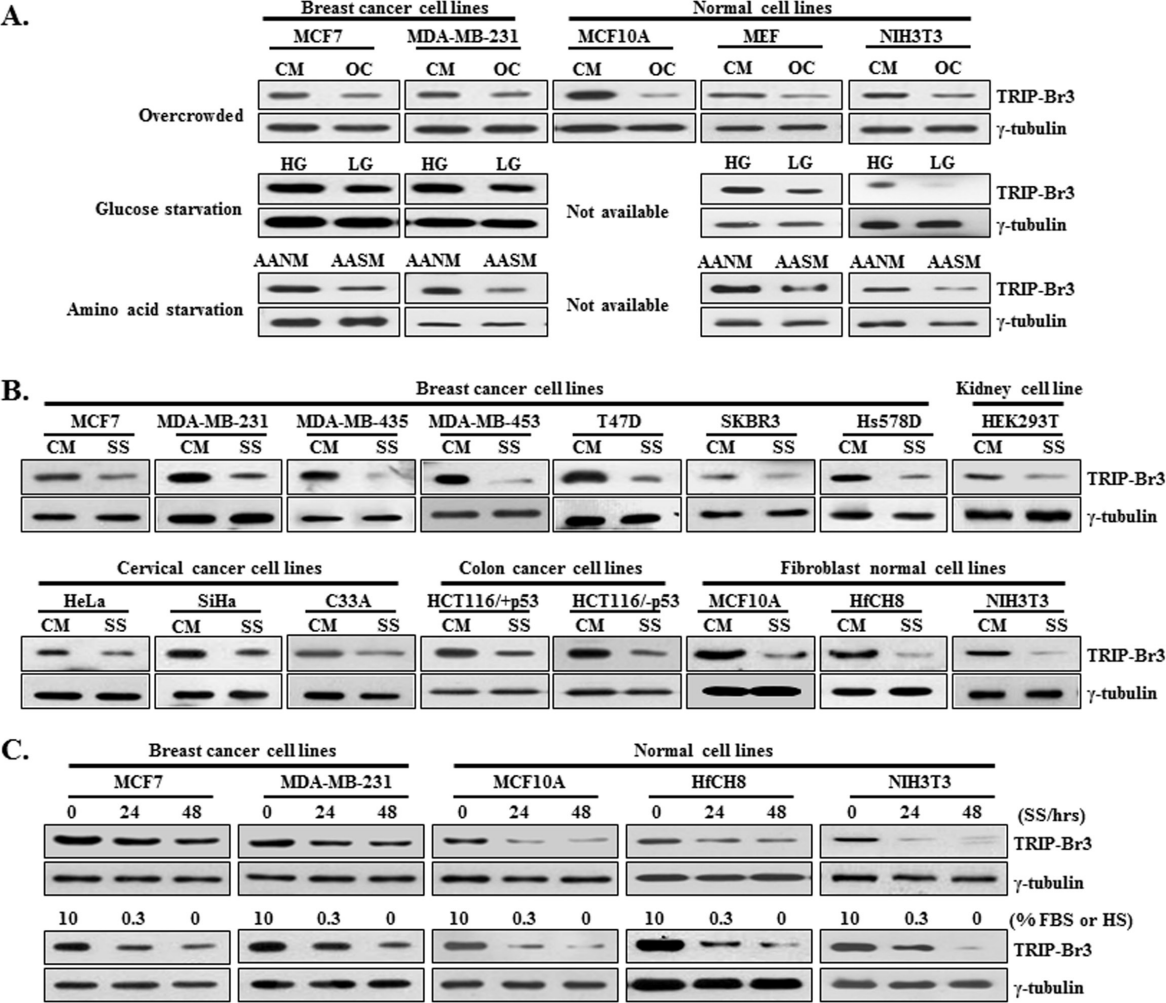
TRIP-Br3 expression levels in cancer and normal cell lines under the stressful condition of nutrient deficiency **(A)** TRIP-Br3 expression levels in breast cancer and normal cell lines under the stressful conditions of nutrient deficiency (overcrowded environment and depletion of glucose or amino acid). Cells were cultured in complete medium until cells reached ~70% confluence (CM) or overcrowded with high levels of cell confluence by culturing in a complete medium for long time (OC). Each cell line was grown in high (HG) or low (LG) levels of glucose containing medium. Cells were also cultured in sufficient amino acid containing normal medium (AANM) or amino acid starved EBSS minimal medium (AASM). **(B)** Cancer and normal cell lines were grown in serum containing complete medium (CM) or serum starved medium (SS). **(C)** Expression levels of TRIP-Br3 in response to serum depletion were measured in time- or concentration-dependent manner.

Low nutrient condition is one of characteristics in cancer cells under overcrowded conditions with high cell density. In particular, highly aggressive and rapidly growing tumors are easily exposed to nutrient deficiency due to uncontrolled growth and proliferation. In the process of finding genes responsible for the resistance to nutrient depletion derived cell death, TRIP-Br3 expression was found to be more decreased in normal cells compared to cancer cells under overcrowded conditions (Figure 1A). To examine what kind of nutrient deficiency is especially responsible for the decreased TRIP-Br1 expression, cells were placed under conditions deprived of representative nutrients, glucose and amino acids, in which TRIP-Br3 expression was decreased significantly in normal cells and slightly in cancer cells (Figure 1A). In particular, serum deficiency, the main stress in this study, markedly decreased TRIP-Br3 protein level in all tested cancer and normal cells (Figure 1B). It was confirmed in time- and dose-dependent ways, in which TRIP-Br3 expression level was more rapidly and greatly decreased in normal cells compared to cancer cells (Figure 1C).

### Mechanisms responsible for the TRIP-Br3 down-regulation during serum starvation

**Figure 2:**
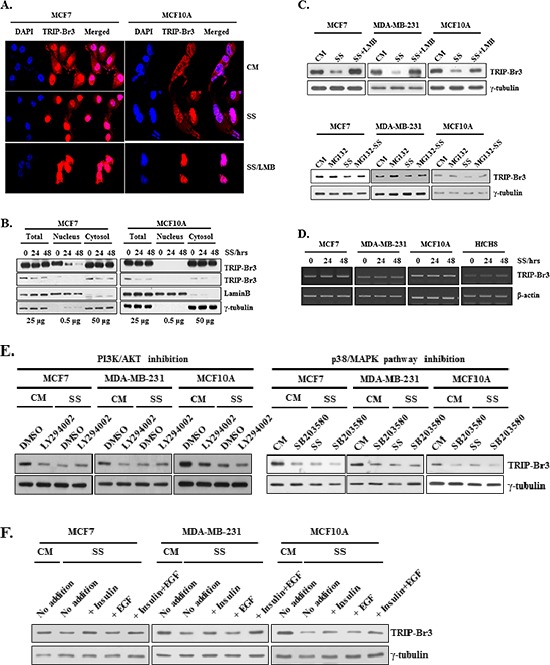
Change of TRIP-Br3 expression level in different sub-cellular localizations under serum starvation **(A)** MCF7 and MCF10A cells were grown in media with or without serum for 48 h. Resulting cells were prepared for immunofluorescence and TRIP-Br3 localization was detected by using confocal microscopy. **(B)** Analysis of TRIP-Br3 location by nuclear fractionation in response to serum starvation for indicated times. Lamin B and γ-tubulin were used as nuclear marker or a loading control, respectively. Loaded amount of proteins are numbered. **(C)** Cells were grown in complete (CM) or serum starved (SS) medium in the absence or presence of 10 ng/ml of LMB for 36 h or 0.1 μM of MG132 for 20 h. Resulting cells were subjected to Western blot analysis. **(D)** TRIP-Br3 gene expression level was checked at the transcriptional level by employing RT-PCR. It was performed after indicated cells were subjected to serum containing complete medium (CM) or serum starved condition (SS) for 12 h or 24 h as mentioned in Methods and Materials. **(E)** Effect of PI3K/AKT and p38/MAPK on TRIP-Br3 expression under the condition of serum deficiencyEach cell line was grown in DMEM or DMEM/F12 with or without 50 μM of LY294002 or 20 μM of SB203580 for 24–48 h in the complete (CM) or serum starved (SS) media. **(F)** Cells were grown in DMEM or DMEM/F12 in the presence or absence of 20 μg/ml of insulin and/or 50 μM of EGF for 48 h in the complete (CM) or serum starved (SS) media.

To elucidate how TRIP-Br3 protein level is decreased under serum starvation, their cellular localization was at first checked. In the complete medium with sufficient serum, TRIP-Br3 was found in both nucleus and cytoplasm of MCF7 cells, but rarely found in nucleus of MCF10A cells (Figure 2A). In the serum starved medium, TRIP-Br3 signals became much weaker compared to those in normal condition (Figure 2A). This was confirmed by employing nuclear fractionation. TRIP-Br3 proteins were significantly decreased in the cytosol and/or nuclei of MCF7 and MCF10A cells in response to serum starvation (Figure 2B). It was proposed that TRIP-Br2 is localized in nucleus but mainly in cytoplasm, in which TRIP-Br2 is transported from nucleus into the cytoplasm through interaction with CRM1 and degraded by proteasome 26S [19, 22]. We therefore suspected that TRIP-Br3 might be transported and degraded in a similar way with that of TRIP-Br2. As expected, Leptomycin B (LMB, an inhibitor of the CRM1-dependent nuclear export pathway) treatment caused TRIP-Br3 to be retained in the nuclei of MCF7 and even MCF10A cells, in which TRIP-Br3 was not degraded (Figure 2A and 2C). In addition, MG132 (proteasome inhibitor) treatment slightly alleviated the TRIP-Br3 down-regulation under serum starvation. In a further study, RT-PCR analysis showed that TRIP-Br3 gene expression was not changed at the transcriptional level (Figure 2D). Taken together, our data suggest that in cancer cells, TRIP-Br3 is found in both cytoplasm and nucleus, in which nuclear TRIP-Br3 is exported to cytosol in a CRM1-dependent way and degraded at least partly by proteasome in response to serum deprivation. In normal cells, TRIP-Br3 presents mainly in the cytosol where cytosolic TRIP-Br3 more rapidly and greatly degraded by proteasome in response to serum starvation.

Next, we tried to find what signaling pathways are involved in the TRIP-Br3 gene expression. Serum contains many different types of survival and growth factors, although its exact and complete composition is still unknown. We suspected that serum starvation-induced TRIP-Br3 regulation might be due to the deficiency of growth and/or survival factors. Many survival and growth factors mediate several signaling pathways through the phosphoinositol-3-kinase (PI3K)/AKT and p38/mitogen-activate protein kinase (MAPK) signaling pathways, which in turn affect gene expression of many genes [3, 7, 8, 31–33]. Therefore, the effect of these signaling pathways were examined using LY294002 (a PI3K/AKT inhibitor) and SB203580 (a p38/MAPK inhibitor). Their inhibition induced TRIP-Br3 down-regulation under normal conditions (Figure 2E). In fact, the effect of serum deficiency on TRIP-Br3 gene expression was slightly reversed by the addition of insulin survival factor but not by EGF (Figure 2F). This data suggest that serum starvation-derived blockage of these signaling pathways is at least partly responsible for the TRIP-Br3 down-regulation during serum depletion.

### Inhibitory effect of TRIP-Br3 on apoptosis in response to serum starvation

**Figure 3:**
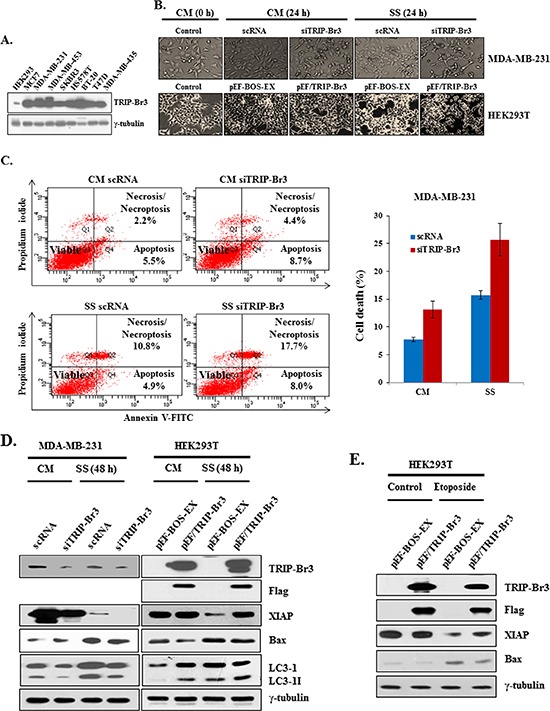
Inhibitory role of TRIP-Br3 in serum depletion induced cell death **(A)** Different expression levels of TRIP-Br3 gene in nine cancer cell lines. **B, C, D, and E**. TRIP-Br3 gene was overexpressed or suppressed by transiently transfecting with TRIP-Br3 silencing RNAs (siTRIP-Br3) or TRIP-Br3 overexpressing plasmid (pEF/TRIP-Br3) with corresponding controls (scRNA or pEF-BOS-EX) as detailed in Methods and Materials. Transfected cells were then incubated in the complete (CM) or serum starved (SS) medium for the indicated times. Microscopic phenotypes of each cell line were photographed **(B)**. Transfected MDA-MB-231 cells were harvested and stained with Annexin V-FITC/propidium iodide for Flow Cytometry Analysis to quantify the serum starvation induced cell death **(C)** Each protein level was checked using Western blot **(D)** HEK293T cells were transfected with TRIP-Br3 overexpressing plasmid (pEF/TRIP-Br3) and then treated with 20 μM of etoposide for 48 h **(E)**.

To elucidate the role of TRIP-Br3 under the nutrient deficient stressful conditions, TRIP-Br3 expression levels were at first compared in nine different cancer cell lines. The highest and lowest TRIP-Br3 levels were detected in MDA-MB-231 and HEK293T cells, respectively (Figure 3A). Therefore, they were chosen for the suppression or overexpression of TRIP-Br3 gene. Interestingly, MDA-MB-231 cells treated with TRIP-Br3 silencing siTRIP-Br3 were much more sensitive to serum starvation-induced cell death compared to control cells, whereas HEK293T cells transfected with TRIP-Br3 overexpressing pEF/TRIP-Br3 vector showed the similar result with control cells (Figure 3B). This data suggest that down-regulation of TRIP-Br3 induces cell death. This conclusion was supported with FACS analysis as shown in Figure 3C. Both in complete or serum starved media, the cell death rates were much higher (~13.1% and ~25.7%) in MDA-MB-231 cells treated with TRIP-Br3 silencing siTRIP-Br3 compared to control cells (~7.7% and ~15.7%).

Next question was how TRIP-Br3 could inhibit cell death. We previously showed that TRIP-Br1 acts as an anti-apoptotic protein by stabilizing the XIAP in breast cancer cells [12]. XIAP, the most potent human IAP protein, is an endogenous cellular suppressor of apoptosis that directly limits the activity of caspases 3, 7, and 9 [34–39]. Based on their similar structures, it was therefore hypothesized that TRIP-Br3 might use similar mechanism in apoptosis. As shown in Figure 3D, XIAP expression was not changed until 24 h but significantly decreased at 48 h in response to serum starvation, which was enhanced by the suppression of TRIP-Br3, but alleviated by TRIP-Br3 overexpression, indicating the positive role of TRIP-Br3 on XIAP expression. It was also confirmed by using another apoptosis inducing drug, etoposide, in which etoposide-mediated apoptosis was also alleviated by TRIP-Br3 overexpression (Figure 3E). In an extended study, role of TRIP-Br3 in the regulation of autophagy was also investigated. Interestingly, the conversion of LC3-I to LC3-II was suppressed by TRIP-Br3 down-regulation but accelerated by TRIP-Br3 overexpression (Figure 3D), implying the positive role of TRIP-Br3 in autophagy. Taken together, our data suggest that serum starvation derived TRIP-Br3 down-regulation inhibits autophagy and accelerates cell death at least by decreasing XIAP expression.

### Positive effect of TRIP-Br1 on XIAP expression under serum starvation

**Figure 4:**
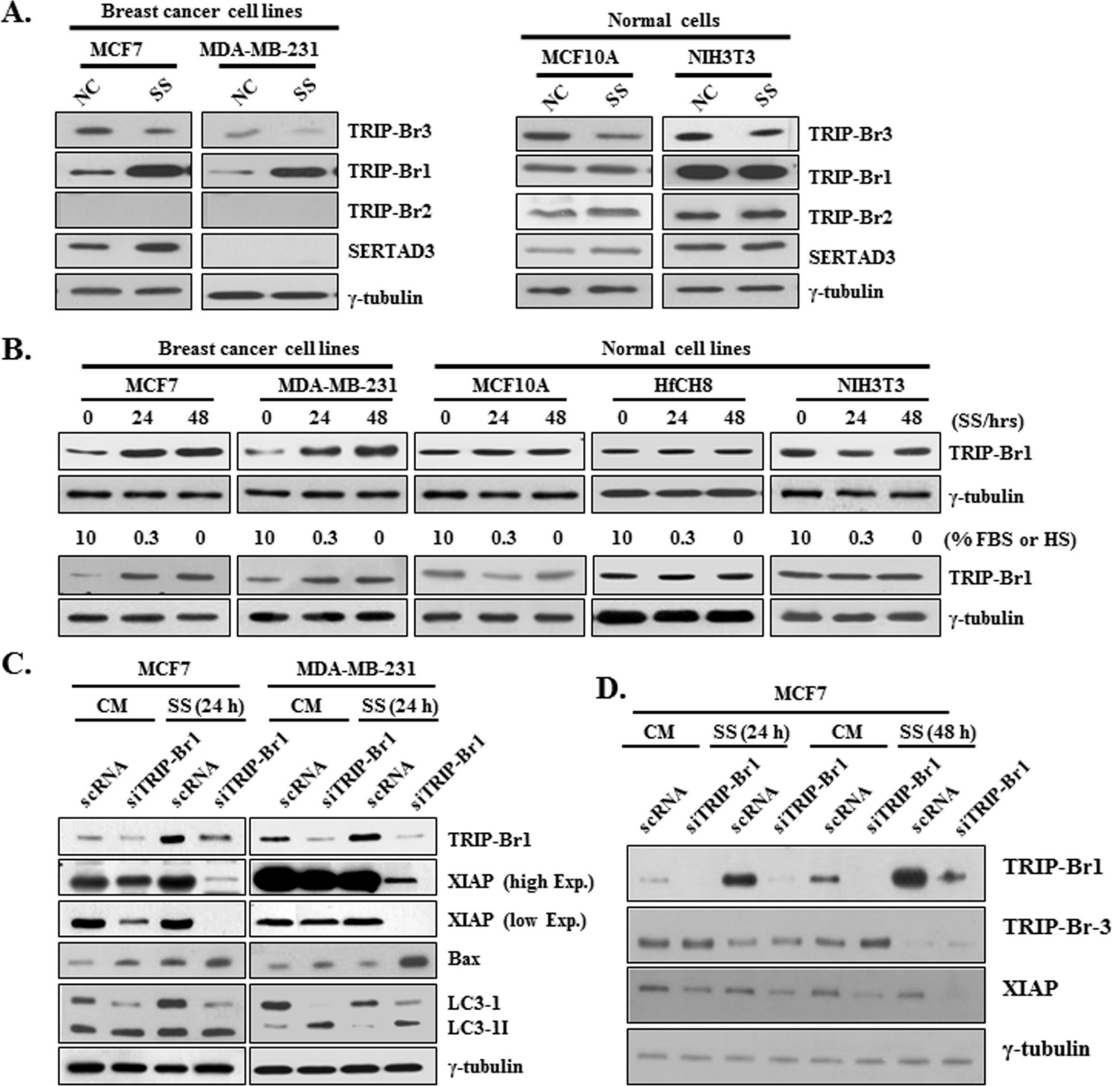
Positive effect of TRIP-Br1 on XIAP expression under serum depletion **(A)** Expression levels of TRIP-Br1, 2, 3 and SERTAD3 proteins were checked in breast cancer and normal cells under the condition of serum deficiency. **(B)** Indicated cells were grown in complete or serum starved media for 24 h or 48 h and the protein levels of TRIP-Br1 were measured by using Western blot. Expression levels of TRIP-Br1 in response to serum depletion were measured in time- or concentration-dependent manner. **(C) and (D)**. TRIP-Br1 genes were suppressed by transiently transfecting with TRIP-Br1 silencing RNAs (siTRIP-Br1) with scRNA control as detailed in Methods and Materials. Transfected cells were then incubated in the complete (CM) or serum starved (SS) medium for the indicated times.

In an extended study, the effect of serum deficiency was also tested in other TRIP-Br family members. Very interestingly, the expression levels of other members were found to be increased in cancer and/or normal cells (Figure 4A). Among them, TRIP-Br1 was especially chosen for further study because its protein level was greatly increased in cancer cells but not in normal cells (Figure 4A). It was confirmed in time- and concentration-dependent ways (Figure 4B). Importantly, TRIP-Br1 also stabilized the XIAP in MCF7 and MDA-MB-231 cells such as the case of TRIP-Br3. As shown in Figure 4C, XIAP down-regulation was enhanced by the suppression of TRIP-Br1. Interestingly, conversion of LC3-I to LC3-II was accelerated by the TRIP-Br1 suppression, suggesting the inhibitory role of TRIP-Br1 in serum starvation–induced autophagy. Effect of TRIP-Br3 and TRIP-Br1 on XIAP expression was further tested in MCF7 cells that both genes were knock-downed, in which XIAP protein level was significantly decreased (Figure 4D). This data suggest that both TRIP-Br3 and TRIP-Br1 exert a positive effect on XIAP expression.

### Stabilization of XIAP through its direct interaction with TRIP-Br3 and TRIP-Br1 and competitive interaction of TRIP-Br3 and TRIP-Br1 to XIAP

**Figure 5:**
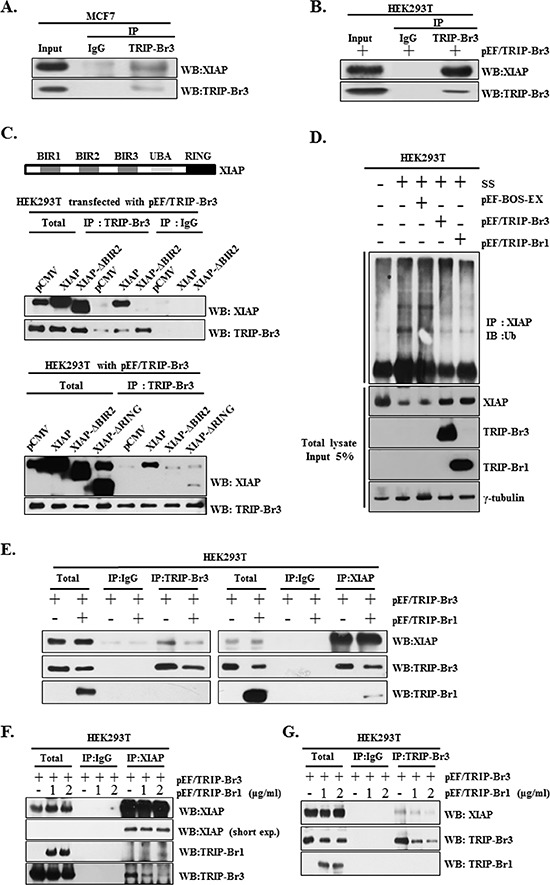
Positive effect of TRIP-Br3 and TRIP-Br1 on the XIAP stability during serum starvation and competitive interaction between TRIP-Br3 and TRIP-Br1 for the binding to XIAP **(A)** Direct interaction between endogenous TRIP-Br3 and XIAP was analyzed by employing immunoprecipitation (IP) and Western blotting (WB) with either anti–TRIP-Br3 or anti-XIAP antibody after MCF7 cells were grown in normal complete medium and harvested at 90% confluence. **(B)** HEK293T cells were transfected with pEF/TRIP-Br3 plasmid and cell lysates were collected for immunoprecipitation analysis. **(C)** Direct interaction of TRIP-Br3 with XIAP through the BIR2 domain. Genomic structure of XIAP consisting of three major domains (three BIRs, UBA, and a zinc-finger RING) is shown. HEK293T cells transfected with pEF/TRIP-Br3 plasmid were co-transfected with control vector (pCMV), flag-tagged full length XIAP (XIAP), BIR2 domain deleted XIAP (XIAP-ΔBIR2), or RING domain deleted XIAP (XIAP-ΔRING). Resulting cells were subjected to Immunoprecipitation assay with anti-TRIP-Br3 antibody. **(D)** Ubiquitination assays were employed after HEK293T cells were transfected with pEF-BOS-EX control vector, pEF/TRIP-Br3, or pEF/TRIP-Br1 vector under normal or serum starved (SS) conditions for 42 h. Cell lysates were immunoprecipitated (IP) with anti-XIAP antibody and immunoblotted (IB) with anti-ubiquitin. The total lysates before immunoprecipitation (Input) and the immunoprecipitate supernatants were then subjected to immunoblotting analysis with corresponding antibodies. **(E)** HEK293T cells were transfected with pEF/TRIP-Br3 and/or pEF/TRIP-Br1 plasmid in complete media for 48 h and the cell lysates were collected for immunoprecipitation analysis. **(F) and (G)** HEK293T cells were co-transfected with pEF/TRIP-Br3 (1 μg/ml) and different concentrations of pEF/TRIP-Br1 plasmids (1 or 2 μg/ml) in complete media for 48 h and the cell lysates were collected for immunoprecipitation analysis. Direct interaction between TRIP-Br3 and XIAP was analyzed by employing immunoprecipitation (IP) with either anti–TRIP-Br3 or anti-XIAP antibodies.

Next question was how TRIP-Br3 can positively affect XIAP expression at the protein level. Our previous study established that TRIP-Br1 inhibits XIAP degradation through a direct association with the BIR2 domain of XIAP (XIAP-BIR2) but no other domains [12]. We therefore tested whether TRIP-Br3 also could stabilize XIAP in a similar way or not. Immunoprecipitation data revealed the direct interaction between endogenous or exogenous TRIP-Br3 and XIAP (Figure 5A and 5B). Furthermore, the deletion of the BIR2 domain abolished the interaction between TRIP-Br3 and XIAP (Figure 5C). This result indicates that TRIP-Br3 directly interacts with XIAP via BIR2 domain. Moreover, deletion of RING domain also weakened the interaction between TRIP-Br3 and XIAP, implying that RING domain is also at least partly responsible for this interaction unlike the case of TRIP-Br1 (Figure 5C). An ubiquitination assay was also performed to determine whether TRIP-BR3 and TRIP-Br1 can inhibit ubiquitination-mediated XIAP degradation under the condition of serum starvation. As shown in Figure 5D, endogenous XIAP was greatly ubiquitinated under the condition of serum starvation compared to the control cells. However, the level of XIAP ubiquitination was significantly reduced in cells transfected with TRIP-Br3 and TRIP-Br1 overexpression vectors. Taken together, our finding strongly suggests that both TRIP-Br3 and TRIP-Br1 bind to and stabilize XIAP by inhibiting its ubiquitination and subsequent degradation. Next question was how TRIP-Br3 and TRIP-Br1 proteins can bind to XIAP, together, or competitively. It was checked by testing the competitive interaction of TRIP-Br3 and TRIP-Br1 with XIAP. HEK293T cells were transiently transfected with pEF/TRIP-Br3 in the absence or presence of pEF/TRIP-Br1 vector. The interaction between TRIP-Br3 and XIAP became weak by TRIP-Br1 overexpression (Figures 5E). It was further confirmed by increasing the concentration of TRIP-Br1 as shown in Figure 5F and 5G. These findings strongly suggest that TRIP-Br3 and TRIP-Br1 seems to compete for the binding to XIAP. In this process, we interestingly found the consistent inverse-relationship in TRIP-Br3 and TRIP-Br1 protein levels. Therefore, we tested whether TRIP-Br1 is responsible for the TRIP-Br3 down-regulation or not. Our unpublished data showed that Doxycycline inducible TRIP-Br1 overexpression very slightly but not significantly decreased TRIP-Br3 expression. However, TRIP-Br1 had no effect on the TRIP-Br3 ubiquitination (data not shown). It could be possible that TRIP-Br1 may affect other proteins or pathways and indirectly cause TRIP-Br3 down-regulation.

### Immunohistochemical expression of TRIP-Br3 in breast tissues

**Figure 6:**
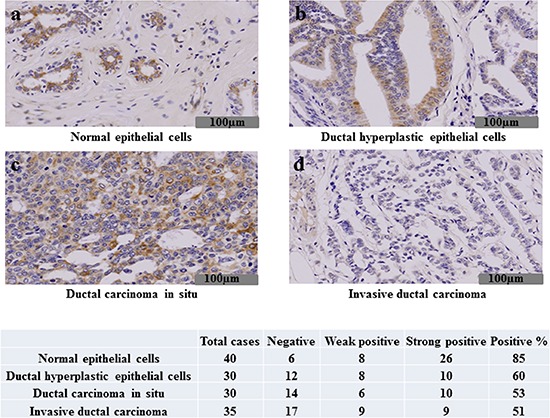
Immunohistochemical expression of TRIP-Br3 in breast tissues Immunohistochemical assay was performed on the normal breast epithelial cells **(A)**, ductal hyperplastic epithelial cells **(B)**, ductal carcinoma *in situ* **(C)** or invasive ductal carcinoma **(D)** as indicated in Materials and Methods. Representative images are shown, in which weak or strong immune-reactivity against TRIP-Br3 are shown in brown color. Forty cases of normal breast tissue, thirty cases of ductal hyperplasia, thirty cases of ductal carcinoma *in situ*, and thirty five cases of invasive ductal carcinoma were tested for the TRIP-Br3 expression. The TRIP-Br3 expression was found in 85% (34/40) of normal breast tissue, 60% (18/30) of ductal hyperplasia, 53% (16/30) of ductal carcinoma *in situ*, and 51% (18/35) of invasive ductal carcinoma. The TRIP-Br3 expression was decreased in the invasive ductal carcinoma compared to the normal breast tissue with statistical significance by student *t*-test (*p* = 0.002).

Our previous data showed that TRIP-Br1 is highly expressed in human breast cancer but weakly in normal tissues, suggesting the role of TRIP-Br1 as an oncoprotein [12]. However, our previous and current data showed that both TRIP-Br3 and TRIP-Br1 have an anti-apoptotic function. Therefore, we further tested whether TRIP-Br3 have a potential to function as a tumor suppressor by employing Immunohistochemistry analysis. Our data revealed that TRIP-Br3 protein was detected to be relatively high in normal tissue samples compared to cancer tissues. Normal epithelial cells have strong positive reaction for TRIP-Br3 in the cytoplasm. Ductal hyperplastic epithelial cells and ductal carcinoma *in situ* also exhibited relatively high level of TRIP-Br3. (Figure 6). However, TRIP-Br3 was not expressed in the invasive ductal carcinoma cells (Figure 6). This data show that TRIP-Br3 protein level might be significantly decreased during breast cancer cell development, implying the role of TRIP-Br3 as a tumor suppressor.

## DISCUSSION

**Figure 7:**
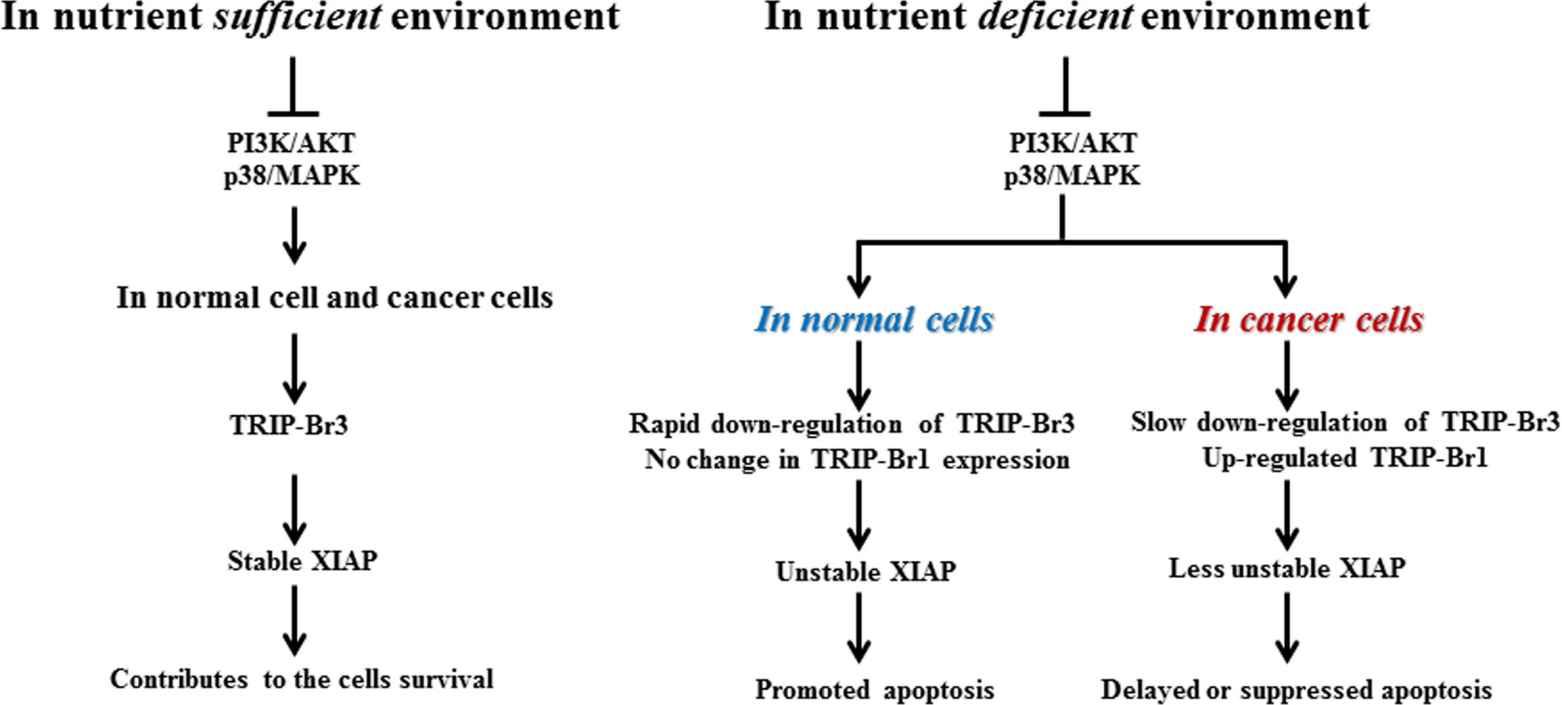
Summary model showing the coordinated regulation by TRIP-Br3 and TRIP-Br1 with anti-apoptotic functions in normal and cancer cells in response to serum deprived condition

Considering all our data, we propose a plausible model, in which TRIP-Br3 and TRIP-Br1 regulate the apoptosis coordinately in normal and cancer cells during serum starvation (Figure 7). In nutrient sufficient environment, TRIP-Br3 contributes the cell survival by stabilizing XIAP. However, this situation can be changed in nutrient deficient stressful environment. In normal cells, cytosolic TRIP-Br3 proteins are rapidly degraded at much earlier times compared to cancer cells. Rapid decrease of TRIP-Br3 triggers XIAP protein to be unstable and eventually lead to cell death. In cancer cells, TRIP-Br3 expression is slightly down-regulated compared to normal cells. Moreover, TRIP-Br3 down-regulation derived XIAP degradation seems to be alleviated by the TRIP-Br1 overexpression.

Our special attention was attracted to the similar but different cellular functions of TRIP-Br3 and TRIP-Br1, in spite of the fact that they belong to the same family. In the effort of finding the reason, we initially compared the amino acid sequences of TRIP-Br3 and other TRIP-Br members. As expected, multiple alignment revealed that TRIP-Br members have a high degree of sequence homology. However, TRIP-Br1 has the highest and lowest homology with SERTADA3 and TRIP-Br3 in sequence similarity, respectively. Biggest difference in amino acid sequences between TRIP-Br3 and other TRIP-Br members was found in NES region. To further examine the homology of TRIP-Br proteins in 3D structure, we built 3D structure of full-length TRIP-Br proteins by using PHYRE2 Protein Fold Recognition Server (data not shown). Our analyses showed that TRIP-Br3 has greatly different structure compared with other family proteins.

In addition, we could not ignore the different results about the functions of TRIP-Br family. Watanabe-Fukunaga *et al* proposed that TRIP-Br1 functions as a tumor suppressor while other researchers consider it as an onco-protein [12, 16, 23–25]. Oue results support that TRIP-Br3 and TRIP-Br1 act as a tumor suppressor or onco-protein in mammalian cells, respectively. At first, our previous Immunohistochemistry analysis showed that TRIP-Br1 is highly expressed in human breast cancer but weakly in normal tissues [12]. However, our current data revealed that TRIP-Br3 protein was detected to be relatively high in normal tissue samples compared to cancer tissues (Figure 7). This data implies the roles of TRIP-Br3 and TRIP-Br1 as a tumor suppressor or an onco-protein and, respectively. At second, our result showed that TRIP-Br3 positively affects autophagy while TRIP-Br1 negatively regulates it during serum starvation. It is now widely accepted that most tumor suppressors activate autophagy, while most oncogenic proteins inhibit it. [40–44]

It has been widely accepted that most tumor suppressor and onco-protein usually induce or inhibit cell death, respectively. However, our result shows that TRIP-Br3 putative tumor suppressor inhibits apoptosis. Subhas *et al* suggested that TRIP-Br1 is required for neuron death, suggesting that TRIP-Br1 promotes cell death [45]. Altogether, we speculate that TRIP-Br3 and TRIP-Br1 may be under the different regulatory control systems in different environments or different types of cells (normal and cancer cells). One possibility is that they may act as an adopter protein and functions differently by changing binding partners. However, this needs to be discovered.

In summary, TRIP-Br3 putative tumor suppressor and TRIP-Br1 onco-protein belong to the same TRIP-Br family with similar genomic structures. However, they exert similar but also very different cellular functions. Rapid down-regulation of TRIP-Br3 accelerated apoptosis in response to serum starvation in cancer and normal cells. However, this was alleviated by TRIP-Br1 up-regulation in cancer cells. Our data also suggest that TRIP-Br3 and TRIP-Br1 respond differently to serum starvation but coordinately regulate apoptosis in cancerous and normal cells by regulating the XIAP stability. Our research about how cancer cells acquire the tolerance to nutrient depletion derived cell death may provide the vital information to cancer research.

Disclosure of potential conflicts of interest: No potential conflicts of interest were disclosed.

## MATERIALS AND METHODS

### Cell lines, cell culture, and used materials

The breast cancer (MCF7, MDA-MB-231, MDA-MB-435, MDA-MB-453, T47D, SKBR3, and Hs578D, and BT-20), kidney cancer (HEK293T), cervical cancer (HeLa, SiHa, and C33A), colon cancer (HCT116/+p53 and HCT116/−p53), human and mouse fibroblast normal cell lines (MEF, HfCH8 and NIH3T3) were cultured in Dulbecco's Modified Eagle's Medium (DMEM; WelGENE Inc., Korea) supplemented with 10% fetal bovine serum (FBS; Gibco BRL, U.S.A) and 1% Antibiotic-Antimycotic (Gibco BRL). MCF10A normal human mammary epithelial cells were grown in DMEM/F12 medium (Invitrogen, Cat. 11330–032, U.S.A) supplemented with 20 ng/ml of epithelial growth factor (EGF; Sigma-Aldrich, Cat. E9644), 100 ng/ml of cholera toxin (Sigma-Aldrich, Cat. C-8052), 10 μg/ml of insulin (Sigma-Aldrich, Cat. I-9278), 0.5 mg/ml of hydrocortisone (Sigma-Aldrich, Cat. H-0888), 5% horse serum (Invitrogen, Cat. 16050–122, U.S.A), and 1% Antibiotic-Antimycotic. All cells were cultured at 37°C in a humidified atmosphere composed of 95% air and 5% CO_2_. HCT116/+p53 and HCT116/−p53 cells were a kind gift from Dr. B. Vogelstein (Johns Hopkins University, U.S.A). SiHa cell line was obtained from the Korean Cell Line Bank (KCLB, Korea (KCLB #30035). Other cell lines were purchased from the American Type Culture Collection (ATCC). LY294004 was purchased from CALBIOCHEM (Cat. 440202, U.S.A), SB203580 and MG132 from Cell Signaling Technology (Cat. 5633 and Cat. 5633, U.S.A), etoposide from A.G Scientific (Cat.E-2004), and LMB from BRP (Cat. BR-C-473, U.S.A).

### Induction of glucose, amino acid, or serum starvation

For the media with high or low glucose level, cells were placed in 4.5 g/L glucose containing DMEM (WelGENE, Cat. LM 001–05, Korea) or glucose-free DMEM (Gibco Invitrogen, Cat. 11966–025, Korea) media with 10% FBS. For amino acid starvation, cells were initially grown in DMEM supplemented with 10% FBS. At 80% confluence, the cells were washed with phosphate buffered saline (PBS) and incubated in Earle's Balanced Salt Solution (EBSS) amino acid-free medium (Gibco Invitrogen, Cat. 14155–063, Korea) containing 5% bovine serum albumin (BSA), 0.1 mg/ml of MgCl_2_, 20 mM of HEPES, and 1% Antibiotic-Antimycotic for 24 h. For serum-sufficient or -deficient conditions, cells were maintained in DMEM or DMEM/F12 media without FBS or horse serum for indicated times, respectively.

### Western blot analysis

Immunoblotting analysis was performed as previously described [29]. Antibodies used in this study were TRIP-Br1 (Enzo Life Sciences, Cat. ALX-804–645), TRIP-Br2 (Abcam, Cat. ab87150), TRIP-Br3 (Abcam, Cat. ab107944), SERTAD3 (Abcam, Cat. ab107728), XIAP (Cell Signaling Technology, Cat. #2042), Bax (SantaCruz Biotechnology, Cat. sc-20067), LC3 (Enzo Life Sciences, Cat. ALX-803–082), LaminB (SantaCruz Biotechnology, Cat. sc-6216), Anti-Flag (Sigma-Aldrich, Cat. F2555), and γ-tubulin (SantaCruz Biotechnology, Cat. sc-7396).

### Reverse transcription (RT)-PCR

RT-PCR analysis was performed as previously described [29]. The oligonucleotide sequences for RT-PCR analysis were pRT-TRIP-Br3-F/R: 5′CTGGTGAAGTTGCAGCTTTG3′/5′GGCAAAGGTCAGAAACTGGA3′; pRT-β-actin-F/R: 5′AGGTCGGAGTCAACGGATTTG 3′/5′GTGATGGCATGGACTGTGGT3′.

### Overexpression or suppression of *TRIP-Br3* and *TRIP-Br1* genes

To establish the TRIP-Br3 and TRIP-Br1 overexpressing cells, cells were transfected with Flag-tagged TRIP-Br3 and TRIP-Br1 overexpressing plasmids (pEF/TRIP-Br3 and pEF/TRIP-Br1) with control vector (pEF-BOS-EX) using Lipofectamine2000 (Invitrogen, Cat. #11668) in Opti-MEM medium (Invitrogen, Cat. 31985). To suppress the TRIP-Br3 or TRIP-Br1, cells were transfected with scrambled small interfering RNA (scRNA), TRIP-Br3 or TRIP-Br1 silencing siRNA (siTRIP-Br3 or siTRIP-Br1). The cells were incubated at 37°C for 6 h and transfection medium was replaced with DMEM without Antibiotic-Antimycotic. pEF/TRIP-Br3 and pEF/TRIP-Br1 plasmids were kindly provided by Dr. Rikiro Fukunaga (Osaka University, Japan). The siTRIP-Br3 and siTRIP-Br1 were purchased from ST Pharm (Korea, Lot#. SCRO-120510–018, 5′GGUGUGUUUUCUUUUGUGCTT3′/5′GCACAAAAGAAAACACACCTT3′) and SantaCruz Biotechnology (Cat. sc-62988), respectively.

### Nuclear fractionation

Nuclear fractionation was performed using NE-PER Nuclear and Cytoplasmic Extraction kit (Thermo Scientific, Cat. 78833, U.S.A) following the manufacturer's instructions.

### Immunofluorescence analysis

MCF7 and MCF10A cells were grown to ~60% confluence on sterilized round type cover glasses in DMEM or DMEM/F12 with or without serum for 48 h. Cells were fixed with 4% formaldehyde in PBS for 15 minutes, washed with PBS, and incubated with TRIP-Br3 (Abcam, Cat. ab107944) primary antibody overnight at 4°C. Cells were then washed with PBS and incubated with Rabbit IgG (H+L) secondary antibody (Jackson Immuno Research Laboratories, Cat. 111–025-003, U.S.A) conjugated to Alex Fluor dyes for 1 h at room temperature in the dark. Cells were washed with PBS and the coverslips were mounted on glass slides with VECTASHIELD anti-fade mounting medium containing 4′6-diamidino-2-phenylindole (DAPI; Vector Laboratories, U.S.A). Samples were imaged using a laser scanning confocal microscope (Leica, Germany) and fluorescence was analyzed.

### Immunohistochemistry (IHC) analysis

IHC data using human tissue samples was kindly provided by Dr. Chang-Jin Kim (Soonchunhyang University Hospital, Korea) and obtained as previously described [29].

### Immunoprecipitation and *in vivo* ubiquitination assay

Cells (2 × 10^7^) were transfected with indicated plasmids. Resulting cells were then harvested and lysed in 200 μl IP buffer A [150 mM of NaCl, 25 mM of Tris-HCl (pH8.0), 1 mM of EDTA, 10% of glycerol, 1% of NP-40, and 1 μl of Protease Inhibitor Cocktail III (A.G. Scientific, Cat. P-1512)] for 30 minutes and each sample was added with 800 μl of IP buffer B [150 mM of NaCl, 25 mM of Tris-HCl (pH8.0), 1 mM of EDTA, and 10% of glycerol]. The lysates were centrifuged at 12,000 rpm for 10 minutes at 4°C. One milligram protein was incubated with TRIP-Br3 (Abcam, Cat. ab107944) or XIAP (SantaCruz Biotechnology, Cat. sc-8789) antibody for 2 h at 4°C. The mixture with protein A/G agarose beads (SantaCruz Biotechnology, Cat. sc-2003) was incubated overnight. The complex was washed with 1 ml of IP buffer C [900 mM of NaCl, 20 mM of Tris-HCl (pH8.0), 1 mM of EDTA, 0.5% of NP-40, and 1 μl of Protease Inhibitor Cocktail III)]. Proteins were eluted from the beads by boiling in SDS sample buffer and analyzed by Western blot with the indicated antibodies. The flag-tagged full length XIAP (XIAP), BIR2 domain deleted XIAP (XIAP-ΔBIR2), or RING domain deleted XIAP (XIAP-ΔRING) plasmids were kindly provided by Dr. Ki-Sun Kwon (Korea Research Institute of Bioscience and Biotechnology, Korea) [30]. In preparation for *in vivo* ubiquitination assays, cells were transfected with indicated plasmids. After immunoprecipitation with an anti-TRIP-Br3 or anti-XIAP antibodies, ubiquitin adducts were detected via immunoblotting analysis using an anti-ubiquitin antibody (SantaCruz Biotechnology, Cat. sc-8017).

### FACS analysis

MDA-MB-231 cells were transfected with scrambled small interfering RNA (scRNA) or TRIP-Br3 silencing siRNA (siTRIP-Br3). Resulting cells were cultured in complete growth medium (CM) or serum free medium (SS) for 48 h. Adherent cells were harvested and washed with PBS twice. Cells (6 × 10^5^) were stained with Annexin V-FITC and Propidium Iodide for 15 minutes according to the protocol of the Annexin V-FITC kit (Enzo Life Sciences, Cat. ADI-ADK-700). The stained cells were washed with PBS buffer once and 800 μl of PBS buffer was added. Resulting cells were then analyzed by using a FACSCanto™ II flow cytometer (BD Biosciences, U.S.A). FITC and propidium iodide emissions were detected in the FL-1 and FL-2 channels by BD FACSDive software (BD Biosciences, U.S.A), respectively. Cell death rate was obtained by recording 1 × 10^4^ cells for each sample and the data is the average of three independent sets.

## Abbreviations

TRIP-Br1 or 3: Transcriptional Regulator Interacting with the PHD-Bromo-domain 1 or 3
XIAP: X-linked inhibitor of apoptosis protein
